# The Effector Cig57 Hijacks FCHO-Mediated Vesicular Trafficking to Facilitate Intracellular Replication of *Coxiella burnetii*

**DOI:** 10.1371/journal.ppat.1006101

**Published:** 2016-12-21

**Authors:** Eleanor A. Latomanski, Patrice Newton, Chen Ai Khoo, Hayley J. Newton

**Affiliations:** Department of Microbiology and Immunology, University of Melbourne at the Peter Doherty Institute for Infection and Immunity, Melbourne, Victoria, Australia; Osaka University, JAPAN

## Abstract

*Coxiella burnetii* is an intracellular bacterial pathogen that infects alveolar macrophages and replicates within a unique lysosome-derived vacuole. When *Coxiella* is trafficked to a host cell lysosome the essential Dot/Icm type IV secretion system is activated allowing over 130 bacterial effector proteins to be translocated into the host cytosol. This cohort of effectors is believed to manipulate host cell functions to facilitate *Coxiella*-containing vacuole (CCV) biogenesis and bacterial replication. Transposon mutagenesis has demonstrated that the Dot/Icm effector Cig57 is required for CCV development and intracellular replication of *Coxiella*. Here, we demonstrate a role for Cig57 in subverting clathrin-mediated traffic through its interaction with FCHO2, an accessory protein of clathrin coated pits. A yeast two-hybrid screen identified FCHO2 as a binding partner of Cig57 and this interaction was confirmed during infection using immunoprecipitation experiments. The interaction between Cig57 and FCHO2 is dependent on one of three endocytic sorting motif encoded by Cig57. Importantly, complementation analysis demonstrated that this endocytic sorting motif is required for full function of Cig57. Consistent with the intracellular growth defect in *cig57*-disrupted *Coxiella*, siRNA gene silencing of *FCHO2* or clathrin (*CLTC)* inhibits *Coxiella* growth and CCV biogenesis. Clathrin is recruited to the replicative CCV in a manner that is dependent on the interaction between Cig57 and FCHO2. Creation of an FCHO2 knockout cell line confirmed the importance of this protein for CCV expansion, intracellular replication of *Coxiella* and clathrin recruitment to the CCV. Collectively, these results reveal Cig57 to be a significant virulence factor that co-opts clathrin-mediated trafficking, via interaction with FCHO2, to facilitate the biogenesis of the fusogenic *Coxiella* replicative vacuole and enable intracellular success of this human pathogen.

## Author Summary

Human Q fever is caused by the intracellular bacterium *Coxiella burnetii*. Successful infection of human cells relies on a Dot/Icm secretion system and the translocation of effector proteins into the host cell cytosol. The functions of many *Coxiella* effector proteins, and their contribution to bacterial growth and host manipulation, remain unknown. We show that a unique effector, Cig57, has an important role in manipulation of host cellular clathrin-mediated trafficking. In particular, Cig57 binds FCHO2, a protein involved in formation of clathrin-coated vesicles, in a manner that is dependent on a tyrosine-based endocytic sorting motif. Through engaging proteins in the clathrin pathway, Cig57 facilitates expansion of the *Coxiella* replicative vacuole and enables the pathogen to replicate to large numbers. Thus, we identify a relationship between a host process and a key virulence protein that are required for pathogen success.

## Introduction

The intracellular bacterial pathogen *Coxiella burnetii* is the causative agent of human Q fever, a zoonotic disease with the potential to cause life-threatening complications. Transmission to humans occurs via inhalation of contaminated aerosols. Human infection can lead to an acute, pneumonia-like illness, or proceed to a chronic disease state in which endocarditis can manifest [1]. During natural infection, *Coxiella* predominantly invades alveolar macrophages, and in order to replicate intracellularly, a spacious and fusogenic lysosome-derived vacuole, termed the *Coxiella*-containing vacuole (CCV), is established by the pathogen. After internalization, *Coxiella* passively traffics through the endolysosomal pathway [2, 3]. The developing vacuole obtains markers typical of early and late endosomes, such as EEA1 and Rab7, and finally matures to a lysosome [4, 5]. Here, with an internal pH of approximately 4.8, and in the presence of proteolytic and degradative enzymes, *Coxiella* becomes metabolically active and will direct the expansion of the CCV before replicating to large numbers [6]. The active form of *Coxiella* is the replicative large cell variant (LCV), distinct from the environmentally stable small cell variant (SCV) [7].

The exact requirements that render the CCV permissive for replication are unknown, however recent mutagenesis studies have demonstrated that a Dot/Icm type IVB secretion system is essential for CCV biogenesis and intracellular replication [8, 9]. This secretion system is activated by the lysosomal environment [10] and more than 130 *Coxiella* effector proteins are known to be translocated from the pathogen into the host cell [8, 11–18]. Multiple mutagenesis studies have identified a small cohort of Dot/Icm effectors that play important roles in CCV biogenesis and intracellular replication of *Coxiella* [14, 16, 19, 20]. However, the function of most of these effectors and why they are required for intracellular success of *Coxiella* remains to be elucidated.

Using HeLa cells as an important model for infection, various host cell vesicular trafficking pathways have been shown to facilitate CCV development and contribute to the infection cycle of *Coxiella*. The retromer trafficking process, required for retrograde transport from endosomes to the *trans*-Golgi network [21], has been shown to contribute to the maturation of the CCV with retromer subunits VPS35 and VPS29 and sorting nexins all required for expansion of the CCV [22]. In addition, v-SNAREs VAMP3, VAMP7 and VAMP8 are found on the CCV membrane and have been shown, via siRNA experiments, to aide fusion events with vesicles in the endolysosomal pathway [23]. The autophagic SNARE, syntaxin-17, is required for the homotypic fusion of CCVs [22, 23]. Indeed, perturbation of autophagy, through both enzymatic inhibition and siRNA treatment of key proteins required for autophagy, results in a multi-vacuole CCV phenotype [20]. *Coxiella*, does not appear to induce host cell autophagy but interaction of the CCV with autophagosomes is demonstrated by the accumulation of LC3 inside the CCV, and Rab27 on the vacuole membrane [20, 24]. This indicates that successful CCVs are most accurately described as autolysosomes [24]. Recently, host cell clathrin-mediated trafficking was also shown to be important for intracellular replication of *Coxiella* [25]. Clathrin is important for endocytosis as well as trafficking events within cells. Clathrin-mediated endocytosis is the process by which host cells internalize material, termed cargo, into clathrin-coated pits, to then be sorted to their subcellular compartments (For a review see [26]). Larson and colleagues showed that siRNA gene silencing of *CLTC* (clathrin) and *AP2B1/AP2M1* (AP-2), but not *AP1*, significantly impeded the intracellular replication of *Coxiella*. The adaptor complex AP-2 acts at the plasma membrane during clathrin-mediated endocytosis, and both AP-1 and AP-3 facilitate clathrin-mediated trafficking from the *trans*-Golgi network [27]. These key proteins, required for endocytosis events at the plasma membrane, facilitate the normal growth of *Coxiella* [25]. Thus, the CCV interacts with multiple host vesicular trafficking processes for successful CCV expansion and intracellular replication.

These host pathways are likely manipulated and controlled by complex interactions with *Coxiella* Dot/Icm effector proteins. For example, initial studies linked the multi-vacuolar CCV phenotype seen in *cig2*::Tn mutants with the multi-vacuolar phenotype seen when silencing host autophagy components [20]. More recently, the effector Cig2 was found to bind phosphoinositide PI(3)P, and this was required for recruitment of autophagy machinery components to facilitate homotypic fusion of CCVs [24, 28]. Additionally, the effector CvpA was found to modulate the association of *Coxiella* with the clathrin trafficking pathway through interaction with AP-2. CvpA is required for intracellular replication of *Coxiella* and this is believed to be linked to the acquisition of endolysosomal lipids and proteins through subversion of the clathrin transport pathway.

Cig57 (CBU1751, a 48.8 kDa protein, 420 amino acids), initially identified as an effector because the promoter region contains a PmrA binding region that indicates that the gene is co-regulated with *i**cm* genes [29], is also required for intracellular replication of *Coxiella* [20]. A transposon-mutagenesis screen to identify novel factors that influence CCV morphology identified multiple transposon insertions, disrupting *cig57*, that caused a significant bacterial growth defect and small vacuole phenotype [20]. Hence this effector plays a vital role in establishing the *Coxiella* intracellular replicative niche. Similar to CvpA, Cig57 contains endocytic sorting motifs that mimic those recognised by adaptor protein complexes in the host cell. In Cig57 there are three endocytic sorting motifs, two dileucine motifs ([DERQ]xxxL[LI]), and one tyrosine motif (YxxΦ), where x represents any amino acid and Φ is a bulky hydrophobic residue [30, 31]. Adaptor protein complexes bind to these motifs usually found on transmembrane proteins such as the transferrin receptor (TfR), to facilitate their selection and uptake into clathrin-coated vesicles (For a review see [30]).

Herein, we examine the function of Cig57 by identifying and examining its interaction with the host protein FCHO2. FCHO2 (88.9 kDa, 810 amino acids) is part of the muniscin subfamily of the EFC domain (extended Fes-CIP4 homology) proteins. Muniscins act at the early stages of clathrin-mediated endocytosis and have been implicated in the initiation of clathrin-coated pits [32, 33]. Specifically, the N-terminal EFC domain of FCHO2 dimerizes and binds the inner plasma membrane, binding to PI(4,5)P2 enriched membranes [33, 34], to enable the curvature seen in the neck of clathrin-coated vesicles. We show FCHO2 is required for optimal CCV formation, and establish that Cig57, interacting with FCHO2 via a tyrosine-based endocytic sorting motif, subverts clathrin to the CCV to facilitate normal vacuole biogenesis and intracellular replication of *Coxiella*.

## Results

### The effector Cig57 interacts with FCHO2

**Fig 1:**
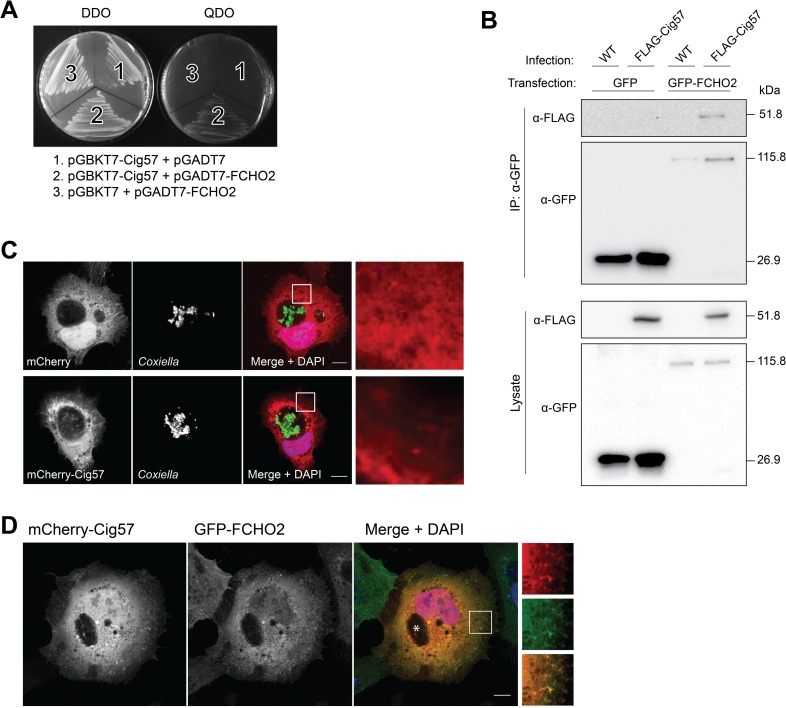
Interaction between Cig57 and FCHO2. **(A)** A yeast-two hybrid screen, using a HeLa cDNA library, revealed FCHO2 as a binding partner of Cig57. Growth on double dropout (DDO) plates for all transformations indicates plasmid retention and yeast viability. Growth on quadruple dropout (QDO) plates indicates the interaction between FCHO2 and Cig57 (segment 2) and the lack of growth in segment 1 and 3 of this plate indicates no interaction with the empty vectors present. **(B)** A Pull-down assay verified the interaction between Cig57 and GFP-FCHO2 during *Coxiella* infection of HEK 293T cells. Cells were infected with wild-type (WT) *Coxiella*, or *Coxiella cig57*::Tn strain expressing 3xFLAG-Cig57 from a plasmid, and transfected with GFP constructs (GFP or GFP-FCHO2). GFP proteins were pulled out using GFP-Trap beads. The presence of 3xFLAG-Cig57 in the pull down lysates indicates interaction between GFP-FCHO2 and Cig57. Results are shown for one repeat of the experiment. **(C)** Representative images showing HeLa cells transfected to express mCherry or mCherry-Cig57 (red) during infection with wild-type *C*. *burnetii* (green). Nuclei are stained in blue with DAPI. Scale bar = 10 μm. **(D)** HeLa cells stably expressing GFP-FCHO2 were transfected with pmCherry-Cig57 and nuclei stained with DAPI (blue). Scale bars represent 10 μm and the CCV is denoted by an asterisk.

To identify potential host protein targets of Cig57, a yeast-two-hybrid (Y2H) assay was performed using the full length Cig57 as bait and a HeLa cDNA library as prey. Cig57 alone did not activate reporter gene expression, as *Saccharomyces cerevisiae* (Y2H Gold, Clontech) carrying pGBKT7-*cig57* could not grow on quadruple dropout (QDO) yeast minimal media (YMM) plates (-Trp, -Leu, -His, -Ade). After screening, only one positive prey clone was identified from the cDNA library, and the insertion was sequenced. This clone encoded amino acids 1 to 433 of the human protein FER/CIP 4 homology only protein 2 (FCHO2). The interaction between Cig57 and FCHO2 1–433 was confirmed by re-transformation of both pGBKT7-*cig57* and pGADT7-*FCHO2* (1–433) into a different *S*. *cerevisiae* strain (AH109). *S*. *cerevisiae* harbouring both pGBKT7-*cig57* and pGADT7-*FCHO2* grew on both double-dropout (DDO, -Trp, -Leu) and QDO YMM agar plates (Fig 1A). No interaction was observed with either pGADT7 or pGBKT7 empty vectors. We next sought to confirm this interaction within mammalian cells using an immunoprecipitation method. Importantly, this was done in the context of infection to address whether the interaction between Cig57 and FCHO2 occurs during infection. HEK 293T cells were infected with *Coxiella* transposon mutant *cig57*::Tn expressing 3xFLAG-Cig57 from a plasmid or WT *Coxiella* as a negative control. These cells were then transfected to express either GFP or GFP-FCHO2. Lysates of infected and transfected cells were incubated with beads that bind GFP, and protein bound to the beads were probed with anti-GFP or anti-FLAG antibodies by Western blot. We detected FLAG-Cig57 in the immunoprecipitate of cells expressing GFP-FCHO2, but not in cells expressing GFP alone, validating the interaction between FCHO2 and Cig57 (Fig 1B). We next sought to determine the intracellular localization of Cig57. In WT infected cells, we transfected mCherry or mCherry-Cig57, and observed that while mCherry had diffuse localization in cells, mCherry-Cig57 is enriched at the CCV and in punctate structures in the cytoplasm (Fig 1C). Because of the interaction with FCHO2, we wanted to determine the relative localization of Cig57 and FCHO2 during infection. We engineered a HeLa cell line that constitutively expresses GFP-FCHO2, and transfected these cells with mCherry-Cig57. FCHO2 was not observed to be enriched at the CCV membrane, however FCHO2 and Cig57 co-localize at punctate structures in the cytoplasm (Fig 1D).

### The Cig57 tyrosine-based endocytic sorting motif is essential for interaction with FCHO2

**Fig 2:**
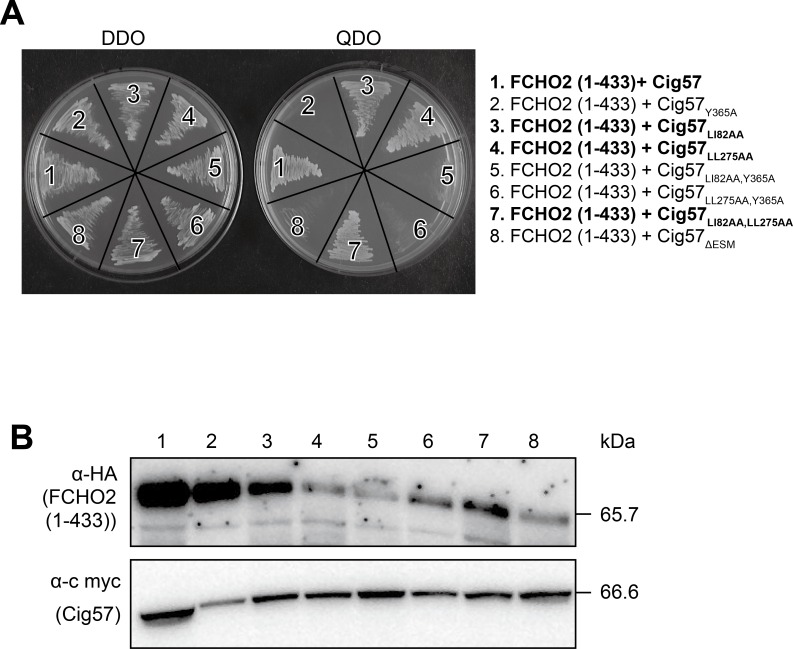
The tyrosine endocytic sorting motif is bound by FCHO2 (1–433). Yeast two-hybrid interactions for co-transformation of pGADT7-*FCHO2* (1–433) and pGBKT7-*cig57*, or the site-directed mutations of Cig57 endocytic sorting motifs as indicated. **(A)** Growth on double dropout (DDO) plates controls for yeast viability and plasmid uptake, and growth on quadruple dropout (QDO) plates indicates an interaction between the indicated proteins. Conditions that indicate protein-protein interactions have been highlighted in bold. **(B)** Immunoblots of yeast cell lysate demonstrate the expression of the proteins. α-HA recognises expression of HA-tagged FCHO2 1–433, 65.7 kDa, from the pGADT7 plasmid, and α-c myc represents expression of tagged Cig57 derivatives (66.6 kDa) from the pGBKT7 plasmid.

Endocytic sorting motifs are typically present on transmembrane receptor proteins, and are recognised by adaptor proteins for selection and sorting of cargo molecules for clathrin-mediated endocytosis [31, 35]. Cig57 contains three predicted endocytic sorting motifs, two of a dileucine type, and one of the tyrosine type. To evaluate the importance of these motifs in Cig57, we created an endocytic sorting motif mutant (ΔESM) construct with mutations in key residues of the predicted Cig57 endocytic sorting motifs (LI82,83AA, LL275,276AA, Y365A), (Cig57_ΔESM_), and evaluated whether this form of Cig57 could bind FCHO2. To investigate whether FCHO2 can recognise these motifs, we transformed yeast with pGADT7-*FCHO2* (1–433) and pGBKT7-*cig57* containing individual mutations in the endocytic sorting motifs, or mutations in combination with each other (Fig 2). We observed growth of all transformants on DDO plates, which indicates yeast viability and successful plasmid uptake, and found no growth on QDO where pGADT7-*FCHO2* (1–433) was transformed alongside pGBKT7-*cig57*_ΔESM_, indicating that one or more of the endocytic sorting motifs are involved in the Cig57-FCHO2 interaction (Fig 2A, segment 8). Individual mutations were also assessed for binding to FCHO2, and we showed that while the dileucine motifs were not important for binding FCHO2, the tyrosine residue Y365, part of the endocytic sorting motif YRKF, is essential for binding to FCHO2 (Fig 2A, segment 2). Hence, we have been able to identify a Cig57 residue essential for this interaction and that FCHO2 might have the capacity to recognise tyrosine-based endocytic sorting motifs. Inability of the proteins to bind to each other was not due to lack of protein expression in the yeast, as all proteins were expressed from the pGAD (anti-HA) and pGBKT (anti-c myc) plasmids (Fig 2B).

### Endocytic sorting motifs encoded by Cig57 contribute to effector function

**Fig 3:**
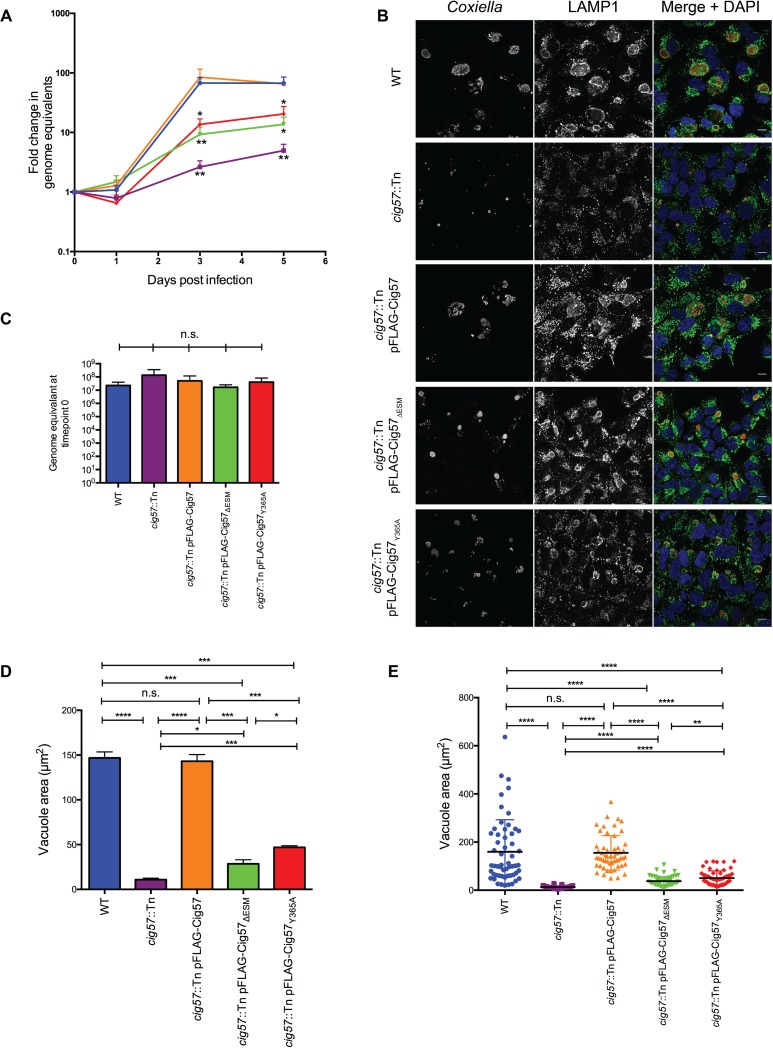
Mutation of Cig57 endocytic sorting motifs disrupts the function of this effector. **(A)** HeLa cells were infected with the indicated strains of *Coxiella* over 7 days and a growth curve was constructed using a quantitative PCR (qPCR) to measure copies of *ompA* and calculating the fold change in genome equivalents (GE) at days 1, 3 and 5 post infection compared to day 0 (n = 3). Error bars represent standard error of the mean (SEM). * = P<0.05, ** = P<0.01 relative to WT growth. **(B)** Confocal immunofluorescence micrographs showing *C*. *burnetii* (red) and LAMP1 (green) at day 3 post infection. Nuclei are stained with DAPI (blue) and scale bar represents 10 μm. **(C)** Genome equivalents recovered at timepoint 0h (4 hours post infection) for each of the indicated strains demonstrates equivalent inoculum and infectivity of the strains. Vacuole sizes were quantified by measuring the area, and results are plotted as **(D)** the mean vacuole area of at least 50 vacuoles for each of three independent experiments and **(E)** all data points from one representative experiment. Error bars represent SEM. * = P<0.05, ** = P<0.01, *** = P<0.001, **** = P<0.0001. n.s. = not significant.

Given the importance of the endocytic sorting motifs, particularly Y365, for binding FCHO2, we examined whether this mutated form of Cig57 could complement the lack of growth seen for the *cig57*::Tn strain. An intracellular growth curve was performed over five days (Fig 3A) which demonstrated the inability of *cig57*::Tn *Coxiella* expressing pFLAG-Cig57_ΔESM_ or pFLAG-Cig57_Y365A_ to grow to similar levels as WT *Coxiella* or *cig57*::Tn pFLAG-Cig57. Interestingly, the ΔESM and Y365A versions of Cig57 are not completely inactive, as growth is not decreased to the same level as the *cig57*::Tn mutant. This phenotype was also observed visually by quantifying and comparing the CCV areas at 72h post-infection from three independent experiments (Fig 3B). Using this measure, vacuole areas formed by the *cig57*::Tn pFLAG-Cig57_ΔESM_ strain (28.5±4.6 μm^2^) are significantly smaller (P = 0.0001) than those produced by WT (146.8±6.7 μm^2^) and the complemented mutant (143.1±7.5 μm^2^) (P = 0.0002). Interestingly, the vacuole sizes of *cig57*::Tn pFLAG-Cig57_Y365A_ (46.9±1.8 μm^2^) were significantly larger than *cig57*::Tn pFLAG-Cig57_ΔESM_ (P = 0.020) which may suggest an additional role for the dileucine endocytic sorting motifs. Importantly, both Cig57_Y365A_ and Cig57_ΔESM_ shows significantly (P = 0.0001 and P = 0.02 respectively) larger vacuoles than *cig57*::Tn mutant (11.0±1.5 μm^2^), indicating that during expression of Cig57_ΔESM_, vacuoles are of an intermediate size (Fig 2C). Analysis of a representative experiment allowed us to visualize that indeed the distribution of vacuole sizes differed between the different strains (Fig 3D). Thus Cig57, and the Cig57 endocytic sorting motifs, particularly the Y365 residue, are important for both intracellular replication of *Coxiella* and expansion of the CCV. To illustrate that all of our *Coxiella* strains were equally as infective, we plotted raw genome equivalent (GE) values for *Coxiella* recovered at timepoint 0h (4 hours post infection), and show that there is no significant difference between the values obtained for each of the strains (Fig 3C).

### Clathrin localizes to the CCV in a manner dependent on Cig57

**Fig 4:**
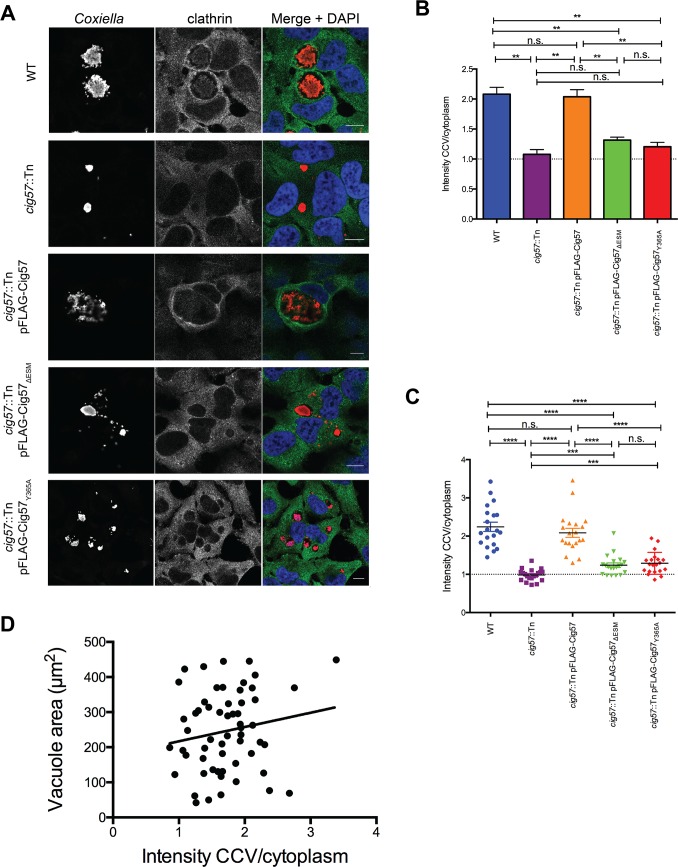
Clathrin localization during *Coxiella* infection. **(A)** Confocal microscopy images of HeLa cells infected for 72 hours with wild type (WT) *C*. *burnetii*, *cig57*::Tn, *cig57*::Tn pFLAG-Cig57, *cig57*::Tn pFLAG-Cig57_ΔESM_ or *cig57*::Tn pFLAG-Cig57_Y365A_. Cells were fixed and stained with anti-clathrin antibody (green), anti-*Coxiella* (red) and DAPI (blue). Scale bar = 10 μm. **(B)** Quantification of clathrin intensity as a ratio of intensity around the vacuole compared to cytoplasmic signal. Fiji was used to sample five signal intensity measurements from both around the CCV and in the cytoplasm for each individual cell allowing calculation of average vacuole and cytoplasmic signal values. A total of 20 cells were measured for each of three independent experiments and error bars represent SEM. **(C)** Representative data points from one experiment in (B). Dotted line at a ratio of 1 indicates no increase in CCV signal compared to the cytoplasm. **(D)** The area of WT vacuoles was plotted against the clathrin intensity ratio, and show a lack of correlation between these two variables.

The distribution of clathrin normally includes plasma membrane and cytosolic puncta throughout the cell [36]. During infection with *Coxiella*, clathrin has been observed around the CCV and this has been shown to require the AP-2 binding effector CvpA [25]. Given that Cig57 interacts with the clathrin related protein FCHO2 we sought to assess the contribution of Cig57 to the accumulation of clathrin on the CCV. HeLa cells were infected and stained for clathrin heavy chain 72 h after infection. Confocal micrographs showed an increased density of clathrin surrounding the CCV in cells infected with WT *Coxiella* and the *cig57*::Tn pFLAG-Cig57 strain (Fig 4A). Importantly, during infection with the *cig57*::Tn mutant strain, or the mutant strain complemented with either pFLAG-Cig57_ΔESM_ or pFLAG-Cig57_Y365A_ clathrin is no longer recruited to the CCV (Fig 4A). When the clathrin signal was quantified, the ratio of signal on the CCV compared to cytoplasmic signal was approximately 2 in WT (2.1±0.1) and the *cig57*::Tn pFLAG-Cig57 strain (2.0±0.1), which was significantly higher than during infection with the mutant (1.1±0.1), the ΔESM complemented mutant (1.3±0.05), or the Y365A complemented mutant (1.2±0.04) (Fig 4B). Datapoints from a representative experiment demonstrate the range of clathrin intensity ratios in a representative infection (Fig 4C). In order to establish whether clathrin recruitment to the CCV was linked to the size of the CCV we examined the relationship between the area of WT *Coxiella* CCVs and clathrin intensity around the CCV, and found no correlation (Fig 4D), with a R^2^ value of 0.029 and a non-significant slope (P = 0.199) deviation from zero. These data implicate Cig57, and its activity mediated by the endocytic sorting motifs, as required for clathrin recruitment to the CCV.

To further validate that clathrin is recruited to CCV membranes, we co-stained infected HeLa cells with LAMP1 and clathrin, and show that when LAMP1 signal is high on the vacuole membrane, clathrin intensity likewise increases (S1 Fig). However this is only true for WT CCVs. Clathrin on *cig57*::Tn vacuoles did not increase at the CCV membrane, denoted by high LAMP1 signal.

### Localization of FCHO2 during infection

**Fig 5:**
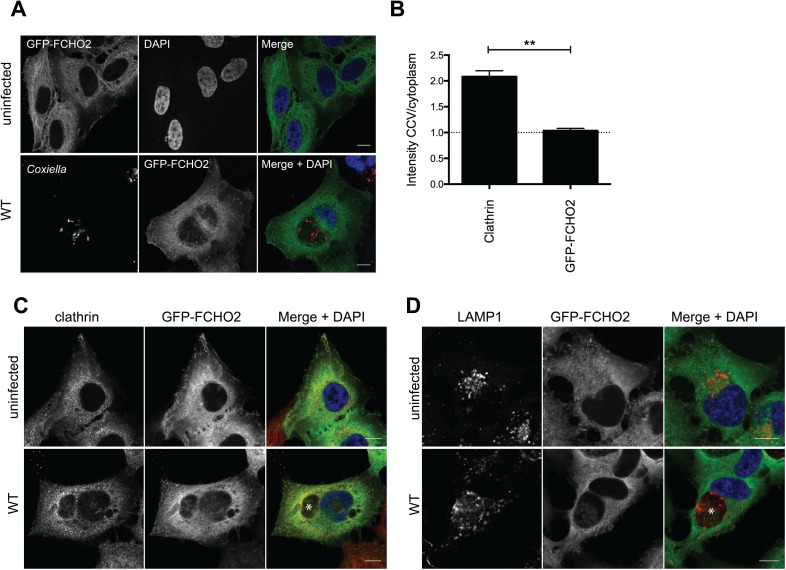
FCHO2 localization during *Coxiella* infection. **(A)** HeLa cells stably expressing GFP-FCHO2 were infected with WT *Coxiella*, or left uninfected. Cells were stained for the bacteria (red) and nuclei (blue, DAPI). Scale bar = 10 μm. **(B)** Quantification of clathrin and GFP-FCHO2 intensity around the vacuole compared to cytoplasmic signal. Error bar represents SEM, and results are representative of three independent experiments. Representative images of uninfected GFP-FCHO2 HeLa cells, or GFP-FCHO2 HeLa cells infected with WT *Coxiella* were stained for **(C)** clathrin (red) or **(D)** LAMP1 (red) and nuclei (blue). Scale bar = 10 μm and CCVs denoted with an asterisk.

Given the CCV localization of clathrin, we next utilized our GFP-FCHO2 stable HeLa cell line to explore the localization of FCHO2. In uninfected cells, FCHO2 localized to the perinuclear region, and the plasma membrane. Likewise, in cells infected with WT *Coxiella*, FCHO2 localization did not change (Fig 5A). Since clathrin was discovered to be on the CCV membrane, we asked whether FCHO2 is also recruited to the CCV. As shown in Fig 5A, and as quantified in Fig 5B, we saw no significant increase in FCHO2 signal on the membrane of the WT CCV. This is despite there being an increased signal of FCHO2 within the region of the CCV, yet there was also an increased FCHO2 signal around the nucleus, thus implying that the FCHO2 signal is not specific to the CCV. To further illustrate that FCHO2 is not recruited to CCVs, we stained infected GFP-FCHO2 cells with clathrin or LAMP1, and note that while clathrin and LAMP1 intensity increases on the CCV membrane, FCHO2 signal does not (Fig 5C and 5D).

### FCHO2 is important for intracellular replication of *Coxiella*

**Fig 6:**
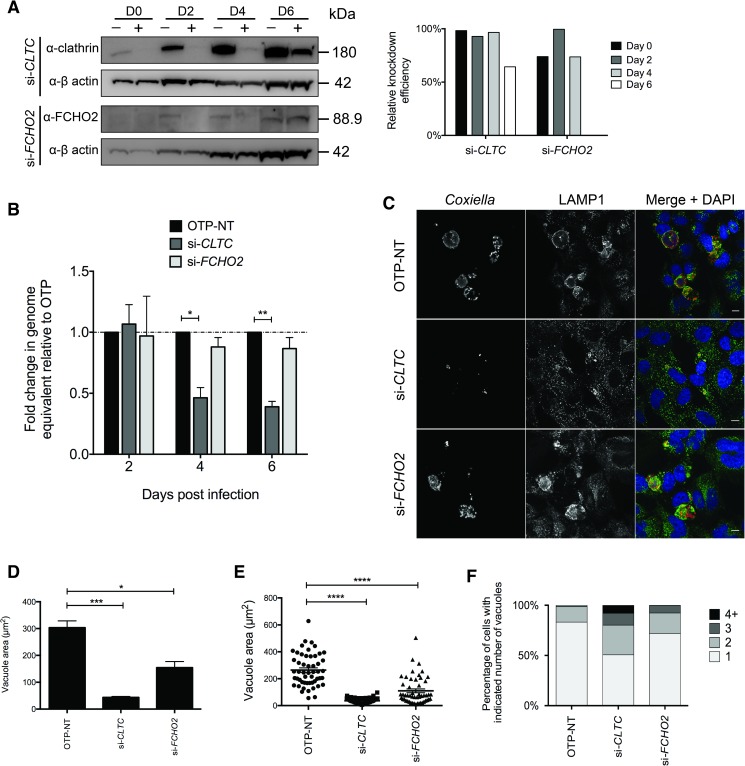
Depletion of FCHO2 or clathrin inhibits *C*. *burnetii* growth. HeLa cells reverse transfected with siRNA against human clathrin heavy chain (si-*CLTC*) and siRNA against human *FCHO2* (si-*FCHO2*) were infected with wild-type *C*. *burnetii* for 2, 4 or 6 days. Non-targeting (OTP-NT) siRNA was used as a control. **(A)** Western blots depict knockdown over the timecourse from the day of infection (D0) to day 6 (D6) and include OTP-NT (–) and targeting (+) samples for each timepoint. β-actin was used as a loading control and the amount of knockdown achieved for each timepoint was graphed relative to the band intensities of β-actin. **(B)** *Coxiella* growth over the timecourse was measured by qPCR. Primers towards *ompA* of *Coxiella* were used to amplify this gene and graph the relative fold growth from the inoculum reading at day 0. Results were then normalized to OTP-NT and graphed relative to the fold change seen in this control. Dotted line corresponds to the fold change of 1.0 relative to OTP-NT and error bars represent SEM. * = P<0.05, ** = P<0.01. **(C)** Immunofluorescent images of the growth of *C*. *burnetii* at 4 days post infection. Cells were stained with anti-*Coxiella* (red) and anti-LAMP1 (green) and the nucleus in DAPI (blue). Scale bars represent 10 μm. **(D)** Images from three independent experiments were used to measure vacuole area during targeting and non-targeting conditions. A total of 50 vacuoles were measured for each condition in each experiment (n = 3). * = P<0.05, *** = P<0.001, error bars = SEM. **(E)** Results of vacuole sizes for one experiment were plotted as individual datapoints (n = 50) to reveal the spread of vacuole sizes seen in each condition. **** = P<0.0001. **(F)** The number of vacuoles per cell were counted in greater than 30 cells per experiment and condition and given a value of either 1, 2 3 or more than 4 vacuoles per cell. Results are displayed as a percentage of the total number of cells counted and represent three independent experiments.

FCHO2 and clathrin are important for normal clathrin-mediated endocytosis. Without clathrin, clathrin-mediated trafficking is blocked. In the absence of FCHO2, endocytosis still progresses, yet clathrin-coated pits are abnormally arranged, with AP-2-positive structures appearing enlarged and clustered [37]. We assessed what the impact of disrupting these genes has on the intracellular replication and CCV formation of *Coxiella*. HeLa cells were transfected with siRNA against clathrin heavy chain (*CLTC*), *FCHO2*, or OnTarget Plus (OTP) non-targeting (OTP-NT). Cells were infected with *Coxiella* 2 days post-siRNA transfection (day 0 post-infection) and lysates were collected for immunoblotting to gauge the level of protein depletion at 0, 2, 4 and 6 days post infection (Fig 6A). The level of knockdown achieved was quantified by measuring band intensities compared to that of the β-actin band intensity. *Coxiella* replication was measured by collecting cell lysates at days 2, 4 and 6 post-infection, and performing qPCR analysis against the *C*. *burnetii ompA* gene to calculate GE. As expected, and described previously, the depletion of cellular clathrin significantly inhibits *Coxiella* growth (Fig 6B and [25]). We compared the level of bacterial growth during silencing of FCHO2 also, and though not statistically significant (P = 0.28), there is a trend towards less bacterial growth when the *FCHO2* gene is silenced (Fig 6B). At day 4 post-infection, samples were fixed for immunofluorescence analysis (Fig 6C) to examine vacuole size during depletion of these key transcripts in clathrin-mediated endocytosis. The area of individual CCVs was quantified and plotted in Fig 6D, in which we show significantly smaller vacuoles when silencing either clathrin (43.4±3.9 μm^2^_,_ P = 0.0005) or FCHO2 (154.5±22.5 μm^2^, P = 0.012), compared to non-targeting conditions (303.4±25.5 μm^2^). Individual datapoints from one representative experiment are plotted in Fig 6E, and show that during silencing of clathrin, vacuoles are uniformly small, and that there is a shift towards smaller vacuoles in FCHO2 silenced cells as noted by the population of smaller CCV areas. These data indicate that FCHO2, as well as clathrin, is required for normal CCV biogenesis. While measuring vacuole sizes, we noted a multi-vacuolar phenotype during silencing of clathrin. Approximately 50% of cells displayed more than one CCV per cell during treatment with *CLTC* siRNA, compared to approximately 15% in non-targeting OTP siRNA (Fig 6F).

Uptake of *Coxiella* is not affected by silencing the clathrin pathway [25]. To corroborate this observation, we evaluated whether the disruption of the clathrin pathway affects the early stages of *Coxiella* infection. HeLa cells were treated with siRNA against *CLTC* or *FCHO2*, and infected for four hours, at which time the cells were fixed and differentially stained for intracellular and extracellular bacteria. There was no difference in the number of intracellular bacteria recovered in either the OTP-NT or silencing conditions (S2 Fig). This indicates that the entry of *Coxiella* is not disrupted in the absence of clathrin or FCHO2.

**Fig 7:**
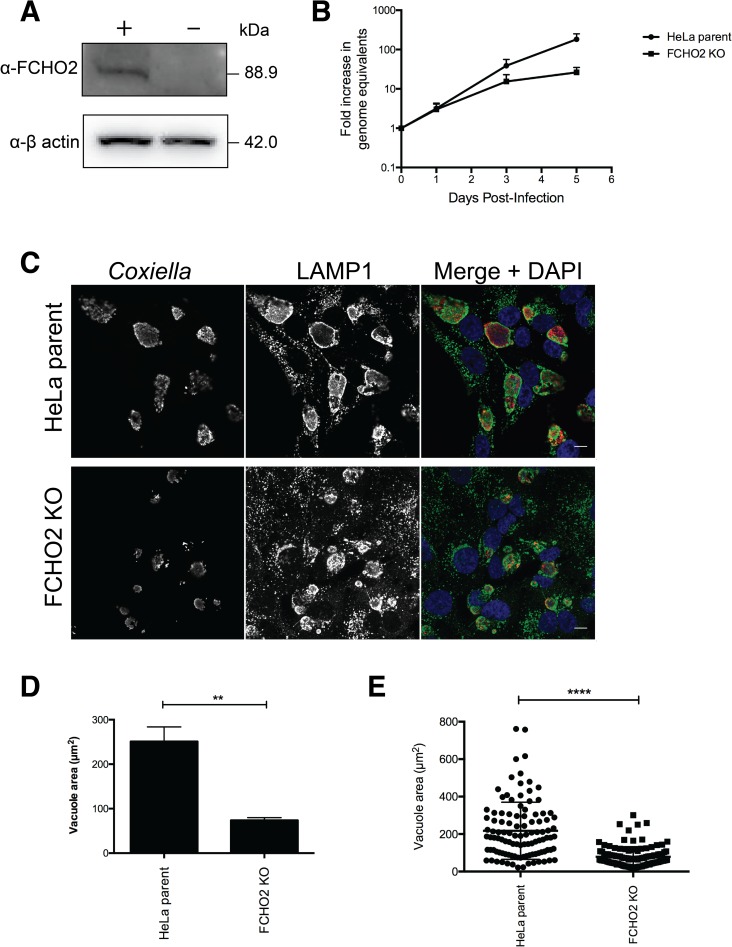
Growth of *Coxiella* is attenuated in cell lines devoid of FCHO2. **(A)** Immunoblots of HeLa cells (+) and FCHO2 knockout (KO) (–) cells using α-FCHO2 and α-β actin antibodies depicting the lack of FCHO2 expression in the KO cell line. **(B)** Genome equivalents of wild-type *Coxiella* were measured by qPCR against the *ompA* gene, in wild-type HeLa parent cells and in FCHO2 KO cells. The fold increase in genome equivalents was measured relative to the day 0 genomic equivalents, taken 4 hours post-infection. * = P<0.05. Results are representative of three independent experiments and error bars represent SEM **(C)** Micrographs of HeLa cells or FCHO2 KO cells at day 3 post-infection with wild-type *Coxiella*. *Coxiella* (red) and LAMP1 (green) were used as markers of the vacuole and host cell nuclei are stained in blue with DAPI. Scale bar = 10 μm. Vacuole areas were measured over three experiments **(D)** and for an individual experiment **(E).** At least 50 vacuoles were measured for each experiment and error bars represent SEM. ** = P<0.01, **** = P<0.0001.

Using siRNA, clathrin was efficiently depleted from the cells until Day 6 however silencing of *FCHO2* at days 4 and 6 post infection was of poor efficiency (Fig 6). This may contribute to the lack of a significant inhibition of *Coxiella* replication and a partial defect in CCV expansion (Fig 6). To overcome the problem of inefficient removal of FCHO2, a HeLa cell line was created which is completely devoid of FCHO2. Using the CRISPR-Cas9 genome editing system, HeLa cells were co-transfected with constructs targeting exon 1 and exon 5 of the *FCHO2* gene, resulting in stable loss of protein production and a FCHO2 knockout (KO) cell line (Fig 7A). These cells, alongside the HeLa parent cell line, were infected with *Coxiella* for 5 days, and the *Coxiella* GE were measured by *ompA* qPCR (Fig 7B). There is a shift towards lower *Coxiella* replication at day 5 post infection in the FCHO2 KO cells compared to the wild-type parent HeLa cell line. At day 3 post-infection, samples were fixed and stained for *Coxiella* and LAMP1 to visualize CCV size (Fig 7C). When quantified, vacuoles are significantly (P = 0.0059) smaller during infection of our FCHO2 KO cell line (73.9±3.5 μm^2^) than when infecting the parental HeLa cell line (251.1±32.9 μm^2^) (Fig 7D). We plotted individual vacuole sizes for one of the three experiments (Fig 7E), to show the distribution of CCV sizes.

### Clathrin recruitment to the CCV is attenuated in the absence of FCHO2

**Fig 8:**
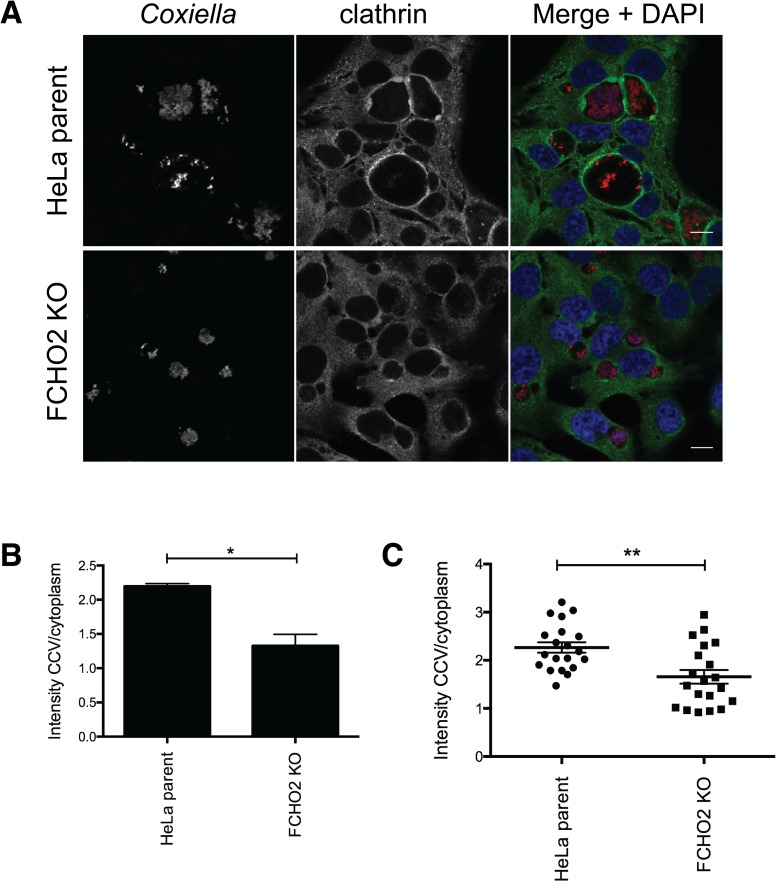
Clathrin localization in FCHO2 KO cells. **(A)** HeLa cells, or HeLa FCHO2 KO cells were infected with WT *Coxiella*, and fixed 72 hours post infection. Coverslips were stained with a clathrin antibody (green) and anti- *Coxiella* (red). Nuclei are stained in blue with DAPI. Confocal micrographs are representative of three independent experiments and the scale bar represents 10 μm. **(B)** Measurements of clathrin intensity were taken around the CCV as well as in the cytoplasm, and a ratio was formed for the intensity CCV/cytoplasm. 20 cells were measured for each of three experiments, and results in **(C)** show all data points from one experiment. Error bars represent SEM and * = P<0.05, ** = P<0.01.

We next asked the question whether clathrin recruitment to the CCV was still able to progress as previously observed in HeLa cells in our FCHO2 KO cells. Using WT *Coxiella*, we infected parental HeLa cells and our FCHO2 KO cell line for three days, and stained for clathrin. As expected, clathrin was found to surround WT vacuoles in HeLa cells, however we observed a lower proportion of FCHO2 KO cells harboured vacuoles that were positive for clathrin (Fig 8A). Again, we measured LAMP1 intensity at a cross section of the vacuole, and show that clathrin increases at the CCV membrane, corresponding to high LAMP1 signal, on HeLa parent CCVs, CCVs formed in FCHO2 KO cells no longer show an increased clathrin intensity at the CCV membrane, where LAMP1 signal is increased (S1 Fig). As in Fig 3, clathrin intensity was approximately double the intensity at the CCV compared to the cytoplasm of wild-type HeLa cells (ratio of 2.2±0.1), however in FCHO2 KO cells, the ratio of clathrin intensity in the CCV compared to the cytoplasm was 1.3±0.3 (Fig 8B). It is to be noted that this phenotype is not as substantial a difference as observed for the CCV clathrin intensity during infection with the *cig57*::Tn mutant, in which the ratio was 1.1±0.1. Indeed, over one representative experiment, we observed a greater range of CCV/cytoplasm ratios during infection of the FCHO2 KO cells (Fig 8C).

## Discussion

The identification and characterization of Dot/Icm effector proteins in *Coxiella* is an active field of research that has been significantly bolstered by the recent advances in axenic culture and genetic manipulation of *Coxiella*. However, ascribing functions to these unique effectors remains challenging. Here, we have identified a host factor and pathway, namely FCHO2 and clathrin-mediated endocytosis, that are targeted by the effector protein Cig57. Clathrin-mediated endocytosis plays an essential role in all nucleated cells. Uptake and recycling of a variety of molecules, from plasma membrane receptors to iron is dependent upon the clathrin pathway. Additionally, clathrin is responsible for the trafficking of early endosomal vesicles to and from the trans-Golgi network playing an important role in delivering cargo proteins to their destination organelles [38, 39].

It is known that clathrin dependent trafficking is essential for the intracellular *Coxiella* lifecycle, as silencing of clathrin results in diminished intracellular *Coxiella* replication ([25] and validated in Fig 6). CvpA, an effector required for intracellular replication of *Coxiella*, was shown to bind AP-2, and now we have shown that another essential effector, Cig57 interacts with a different component of clathrin-coated vesicles, FCHO2 [25]. FCHO2 belongs to the muniscin family of proteins, which also includes FCHO1 and SGIP1 [40]. FCHO1 and 2 are thought to be involved in initiating clathrin-mediated endocytosis, though this is debated in the field as clathrin-mediated endocytosis still occurs in the host in the absence of FCHO1/2 albeit with abnormal morphology [33, 37, 41]. Nevertheless, FCHO2 arrives early to the site of clathrin-mediated endocytosis and aids in sculpting the plasma membrane to form the spherical clathrin-coated vesicles [32, 34]. These proteins contain an N-terminal EFC domain responsible for membrane binding, dimerization and induction of membrane curvature, alongside a linker region, followed by a C-terminal μ-homology domain. The μ-homology domain is an interaction hub, facilitating binding to the clathrin accessory proteins EPS15 and intersectin [33]. The interaction we observed using the yeast two hybrid system with FCHO2 was restricted to the N-terminal region (amino acids 1–433), indicating that Cig57 must bind within the EFC domain or the linker region. Binding the EFC domain may mean Cig57 is altering the membrane-binding capacity of FCHO2, by either blocking its ability to dimerize or bend membranes, or possibly even post-translationally modifying the protein in this region.

We have shown that FCHO2 is required for normal CCV biogenesis during infection with *Coxiella*. CCVs are significantly smaller in FCHO2 KO HeLa cells and there is a trend towards reduced replication of *Coxiella* in these cells. Additionally, the *Coxiella* growth defect in the absence of FCHO2 is not as severe as the growth defect in the absence of clathrin. Taken together, this indicates a level of dependence on the clathrin-mediated pathway for growth of *Coxiella*, where the extent of intracellular bacterial growth may be proportional to the amount of endocytosis exhibited.

FCHO1 is a close homologue of FCHO2. They share approximately 50% homology overall at the protein level. However, the amount of FCHO1 present in HeLa cells is very low, such that endogenous FCHO1 is undetectable by Western Blot analysis [32, 37, 41]. This study has not determined whether Cig57 has the same affinity for FCHO1 as it does FCHO2, but if it does, there is a potential for there to be a greater growth defect in the absence of both FCHO1 and FCHO2. We did not pursue this in our study as it has been previously shown that a FCHO1/2 double knockout is indistinguishable from a FCHO2 knockout [37]. The formation of clustered and abnormal clathrin-coated vesicles is equivalent in both cases.

Interaction with FCHO2 occurs through the Cig57 tyrosine-based endocytic sorting motif. To our knowledge, this is the first report of the ability of FCHO2 to recognise such motifs, as they are normally recognised by adaptor protein complexes such as AP-2. Indeed, CvpA also contains endocytic sorting motifs and these mediate interaction with AP-2 [25]. Whether Cig57 is also able to simultaneously bind AP-2 will be an interesting line of further research. Complementation of *cig57*::Tn with *cig57* encoding mutated endocytic sorting motifs leads to CCVs that phenocopy the small vacuole phenotype observed in FCHO2 KO cells (Fig 7). Hence, the inability of the Cig57_ΔESM_ and Cig57_Y365A_ to complement the *cig57*::Tn mutant is likely due to the inability of this modified effector to bind FCHO2. Importantly, these strains were not attenuated to the same levels as the *cig57*::Tn mutant which may indicate that Cig57 possesses other activity and perhaps the interaction between Cig57 and FCHO2 acts to facilitate Cig57 activity on other components of the clathrin machinery. Interestingly, our results show that the Y365A mutation of Cig57 is not as attenuating as the ΔESM combined mutations, particular in relation to the expansion of the CCV. This may suggest a further role for the dileucine endocytic sorting motifs in full Cig57 function.

Clathrin-coats are formed at the plasma membrane, at the *trans*-Golgi network or on endosomes, and associate with adaptor protein complexes for selection of protein or lipid cargo and specificity of the final destination. Vesicle budding and receptor sorting are facilitated by the formation of a clathrin coat at a membrane. During infection with *Coxiella* the final destination of some of the clathrin-coated vesicles appears to be the CCV, as evidenced by the accumulation of clathrin on the CCV (Fig 5 and [25]). This would offer advantages to *Coxiella* to sequester extra membrane from the clathrin-coated vesicles, and to enable the delivery of nutrients in the form of cargo proteins and lipids from the vesicles to the CCV. The recruitment of clathrin-coated vesicles is dependent on the interaction between Cig57 and FCHO2. *Coxiella* lacking Cig57 cannot recruit clathrin to the CCV and similarly, clathrin recruitment is diminished in the absence of FCHO2. Overall this leads to biogenesis of a CCV that has reduced ability to expand and support *Coxiella* replication.

Despite the requirement for FCHO2 to promote the recruitment of clathrin on CCV membranes, we did not observe recruitment or enrichment of FCHO2 on the CCV. This does not rule out the possibility that this host protein is dynamically cycling on and off the CCV membrane. Importantly, FCHO2 is also not observed on mature clathrin-coated vesicles as it acts early to in the initiation of endocytosis. Thus, the Cig57-FCHO2 interaction may occur at the plasma membrane or other compartments in the cell where FCHO2 has been observed, likely due to the affinity of FCHO2 to particular phospholipids [32]. We therefore cannot discount that Cig57 is taking advantage of an as yet undiscovered role that FCHO2 has in the host. Further investigation of the biochemical function of Cig57 and investigating the functional outcome of the Cig57-FCHO2 interaction will be an exciting area of future study. This line of research will likely reveal the complex mechanisms employed by *Coxiella* to establish a unique intracellular niche to support replication and virulence within the human host.

## Materials and Methods

### Cell culture

Bacterial strains and plasmids used in this study are listed in S1 File. *Coxiella burnetii* Nine Mile Phase II (NMII), strain RSA439 was cultured axenically in liquid ACCM-2 as previously described [42]. Kanamycin and/or chloramphenicol were added to ACCM-2 at 300μg/ml and 3μg/ml respectively when required. *E*.*coli* XL1-Blue or DH5α were cultured in Luria-Bertani medium. Yeast strains were grown at 30°C in YPD (yeast extract/peptone/dextrose) or YMM (yeast minimal media) supplemented with 2% glucose and amino acids including methionine (20 μg/ml), adenine (20 μg/ml), histidine (20 μg/ml), uracil (20 μg/ml) tryptophan (20 μg/ml) and leucine (30 μg/ml) when necessary. HeLa human cervical epithelial cells (CCL-2; ATCC, Manassas, VA), and human embryonic kidney (HEK) 293T cells (a gift from Elizabeth Hartland’s laboratory, University of Melbourne) were cultured in Dulbecco’s Modified Eagle’s Media (DMEM) GlutaMAX (Gibco) supplemented with 10% heat inactivated foetal bovine serum (FBS) at 37°C with 5% CO_2_

### Cloning and plasmids

Plasmid DNA was isolated using the QIAprep spin miniprep kit (Qiagen), and bacterial or HeLa gDNA was isolated using the Zymo gDNA extraction kit. Oligonucleotides to amplify gene products were obtained from Sigma, and are listed in S1 File. DNA modifying enzymes were obtained from NEB and used according to standard procedures.

### CRISPR-Cas9 genome editing

pSpCas9(BB)-2A-Puro (pX459) V2.0 was a gift from Feng Zhang (Addgene plasmid #62988). Guide RNA specific for regions within *FCHO2* (designed using http://crispr.mit.edu/, see S1 File) were cloned into pX459 as previously described [43]. HeLa CCL2 cells were seeded at 2.5 × 10^5^ in 6-well dishes and the following day co-transfected with a total of 2500 ng DNA specific to two exons using Lipofectamine 3000 according to the manufacturer’s protocol. Cells were selected with puromycin (5 μg/ml) and clonally selected in 96-well plates. Modification of *FCHO2* resulting in no protein production was confirmed by Western Blot analysis as below.

### Creation of GFP-FCHO2 stable cell line

FUGW was a gift from David Baltimore (Addgene plasmid #14883). HEK 293T cells were seeded into a 10cm dish and transfected with 7.5 **μ**g pFUGW-GFP-FCHO2, 3.5 **μ**g pPAX and 2.5 **μ**g pVSV-G for 48 hours with Lipofectamine 3000. Lentiviral particles were collected, filtered through 0.45 **μ**m and used to infect HeLa cells in 6-well plates for 48 hours as previously described [44].

### Yeast 2-Hybrid

For screening, Cig57 from *Coxiella* was used as bait. The Matchmaker pre-transformed HeLa cDNA library (Clontech) was mated with *S*. *cerevisiae* carrying pGBKT7-*cig57* according to manufacturer’s protocols (Clontech PT3183-1 manual). The Y2H Gold or AH109 strains were co-transformed with the relevant pGBKT7 or pGADT7 plasmids using the lithium acetate method and plated on DDO (-Trp, -Leu) or QDO (-Trp, -Leu, -His, -Ade) YMM plates.

### Western blot analysis

Samples were suspended in 4x NuPAGE LDS sample buffer (Life Technologies) containing 50μM DTT. Proteins were separated with NuPAGE 4–12% Bis-Tris gels (Life Technologies) and transferred to PVDF membranes using the iBLOT-2 (Life Technologies). Membranes were blocked in 5% skim milk in Tris buffered saline containing 0.1% Tween 20 (TBST) and antibodies were diluted in 5% BSA or skim milk in TBST.

### Fluorescence microscopy

Cells were fixed with 4% paraformaldehyde for 20 minutes at room temperature and permeabilized with 0.05% saponin and 2% BSA in PBS (blocking solution). Primary and secondary antibodies were diluted in blocking solution and DAPI was diluted in PBS before coverslips were mounted using Prolong Gold Antifade (Invitrogen). Images were acquired with a Zeiss LSM700 or LSM710 confocal laser scanning microscope and processed using Fiji [45]. To measure clathrin intensity, five measurements of intensity were taken at the CCV or in the cytoplasm and averaged. For immunofluorescence microscopy (IF) and western blotting (WB) the following antibodies were used at the designated dilutions: α-clathrin heavy chain (Abcam, WB = 1:2000, IF = 1:1000), α-FCHO2 (ThermoFisher, WB = 1:1000), α-β-actin (Perkin Elmer, WB = 1:5000), polyclonal α-*Coxiella* (IF = 1:10000), α-LAMP1 (DHSB, IF = 1:250), HRP-conjugated goat α-mouse (BioRad, WB = 1:3000), HRP-conjugated goat α-rabbit (Perkin Elmer, WB = 1:3000), AlexaFluor 488 (ThermoFisher, IF = 1:2000), AlexaFluor 568 (ThermoFisher, IF = 1:2000). For clathrin localization studies, HeLa cells were seeded at 2.5 × 10^4^ per well in 24 well plates with coverslips and the following day infected at an MOI of 100. Cells were incubated for 72 hours and processed as described above.

### Intracellular *Coxiella* growth curves

In 24-well plates, HeLa cells were seeded at 5×10^4^ per well and infected with the relevant *Coxiella* strains the following day at an MOI of 50. To calculate the MOI, *Coxiella* was quantified using the Quanti-iT PicoGreen dsDNA Assay kit (Life Technologies) [46]. Cells were incubated for 4 hours at 37°C before being washed once with PBS and the media replaced with DMEM containing 5% FBS. At this time (day 0), as well as 1, 3 and 5 days post infection, cells were lysed in dH_2_O and pelleted by centrifugation. Genomic DNA was extracted from the samples and genome equivalents (GE) were quantified by qPCR specific for the *Coxiella ompA* gene [47]. Samples were collected for immunofluorescence analysis at day 3 as described above.

### RNA interference

HeLa cells were transfected with small-interfering RNA (siRNA) using siGenome SMARTpools (Dharmacon, GE Life Sciences) against human *CLTC* (M-004001-00) and *FCHO2* (M-024508-01) or with ON-TARGETplus (OTP) Non-targeting pool (D-001810-10-05) using Dharmafect-1 (Dharmacon, GE Life Sciences). Cells were seeded at a density of 2.5×10^5^ in 6-well plates with a final concentration of 50 nM siRNA. After a two-day incubation, cells were replated at a density of 1.0×10^4^ in 24-well plates, and simultaneously infected with *Coxiella* NMII at an MOI of 10 by centrifugation at 500 × g for 30 minutes. Cells were washed once with PBS and media replaced with DMEM containing 10% FBS. This timepoint was designated day 0. Cells were processed for western blot analysis, genome quantification and immunofluorescence as described above at days 2, 4 and 6 post infection.

### Pull down assay

Persistently infected HEK 293T cells in 10 cm dishes were transfected for 48 hours with GFP constructs using FuGENE6 before lysing in lysis buffer (10mM Tris (pH 7.5), 150 mM NaCl, 0.5 mM EDTA, 0.5% NP-40) for 30 minutes on ice. Lysates were clarified by centrifugation at 17000 x g and 10% collected for input analysis. The remaining lysate was added to 20 μl of GFP beads (ChromoTek) and incubated with mixing at 4°C for four hours. Beads were washed extensively with wash buffer (10mM Tris (pH 7.5), 150 mM NaCl, 0.5 mM EDTA, 0.01% NP-40) before being resuspended in LDS sample buffer and boiled for 10 minutes.

### Statistical analysis

Statistical analyses were performed with Prism (GraphPad Software, Inc.) by use of the unpaired Student’s *t* test. P values less than 0.05 were considered to be significant.

## Supporting Information

S1 Fig LAMP1 localisation validates the recruitment of clathrin to the CCV membrane. HeLa cells were **(A)** infected with WT *Coxiella* or *cig57*::Tn mutant *Coxiella*, or **(B)** HeLa parent and FCHO2 KO cells were infected with WT *Coxiella* and stained with clathrin (green) and LAMP1 (red). Nuclei are blue with DAPI. Arrow indicates the cross section at which the intensities are plotted for LAMP1 and clathrin (right). Scale bar = 10 μm.
(TIF)

S2 Fig Entry of *Coxiella* is not affected by clathrin or FCHO2. HeLa cells were treated with siRNA against *CLTC* or *FCHO2* **(A)** or HeLa cells and FCHO2 KO cells were seeded **(B)** and infected with WT *Coxiella* for four hours, at which point a 0h timepoint for GE was taken. Plotted are the raw values of GE at 0h. Error bars represent SEM for three independent experiments. **(C)** HeLa cells were subjected to silencing of *CLTC* or *FCHO2* or **(D)** HeLa and FCHO2 KO cells were infected with WT *Coxiella* for 4 hours, at which point the cells were fixed and stained for intracellular and extracellular bacteria. Shown is the percentage of bacteria which were internalised at this timepoint. Error bars represent SEM and results are representative of three independent experiments.
(TIF)

S1 File List of strains, plasmids and oligonucleotides used in this study. (XLSX)
