# Supplementary figures and images for: The Effector Cig57 Hijacks FCHO-Mediated Vesicular Trafficking to Facilitate Intracellular Replication of Coxiella burnetii
Source: PLoS Pathog. 2016 Dec 21;12(12):e1006101. doi: 10.1371/journal.ppat.1006101 (PMC5176192; doi:10.1371/journal.ppat.1006101)

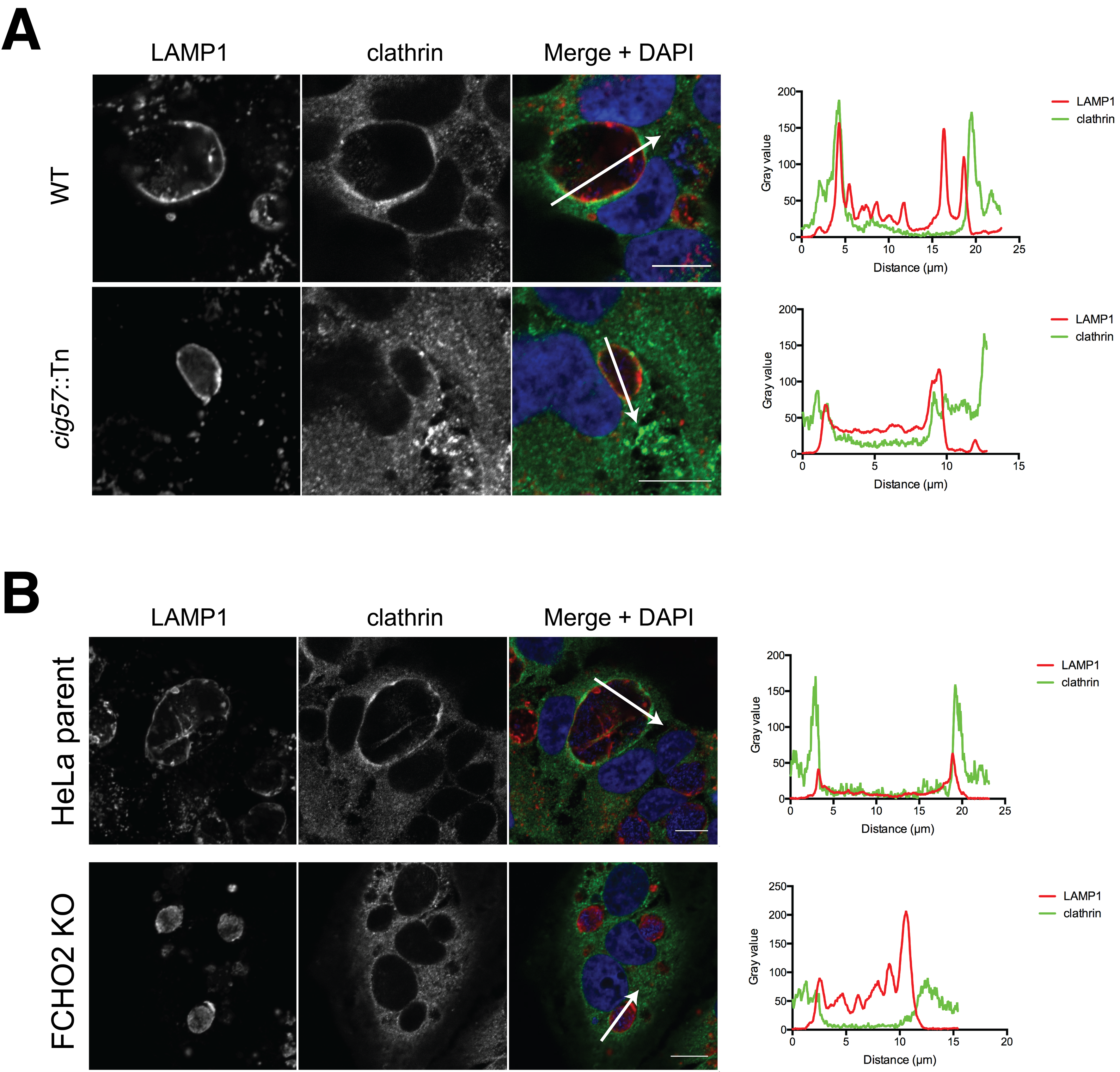

Supplement: S1 Fig — HeLa cells were (A) infected with WT Coxiella or cig57::Tn mutant Coxiella, or (B) HeLa parent and FCHO2 KO cells were infected with WT Coxiella and stained with clathrin (green) and LAMP1 (red). Nuclei are blue with DAPI. Arrow indicates the cross section at which the intensities are plotted for LAMP1 and clathrin (right). Scale bar = 10 μm. (TIF) [file ppat.1006101.s001.tif]

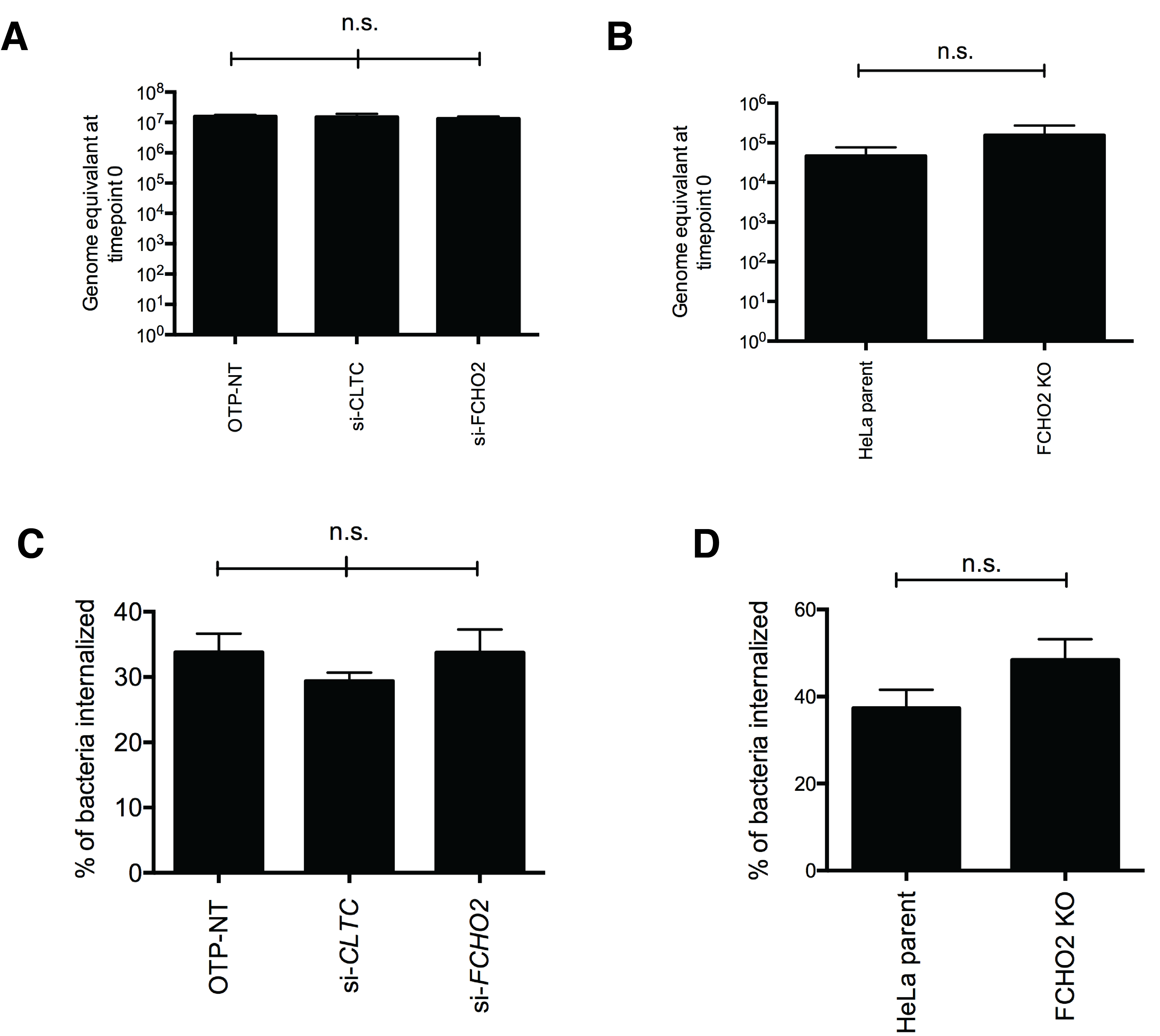

Supplement: S2 Fig — HeLa cells were treated with siRNA against CLTC or FCHO2 (A) or HeLa cells and FCHO2 KO cells were seeded (B) and infected with WT Coxiella for four hours, at which point a 0h timepoint for GE was taken. Plotted are the raw values of GE at 0h. Error bars represent SEM for three independent experiments. (C) HeLa cells were subjected to silencing of CLTC or FCHO2 or (D) HeLa and FCHO2 KO cells were infected with WT Coxiella for 4 hours, at which point the cells were fixed and stained for intracellular and extracellular bacteria. Shown is the percentage of bacteria which were internalised at this timepoint. Error bars represent SEM and results are representative of three independent experiments. (TIF) [file ppat.1006101.s002.tif]
